# Development and validation of supervised machine learning multivariable prediction models for the diagnosis of *Pneumocystis jirovecii* pneumonia using nasopharyngeal swab PCR in adults in a low-HIV prevalence setting

**DOI:** 10.1093/inthealth/ihae052

**Published:** 2024-08-29

**Authors:** Rusheng Chew, Marion L Woods, David L Paterson

**Affiliations:** Mathematical and Economic Modelling Department, Mahidol Oxford Tropical Medicine Research Unit, c/o Faculty of Tropical Medicine, Mahidol University, 3rd floor, 60th Anniversary Chalermprakiat Building, 420/6 Ratchawithi Road, Ratchathewi, Bangkok 10400, Thailand; Centre for Tropical Medicine and Global Health, University of Oxford, Oxford OX3 7LG, UK; Faculty of Medicine, University of Queensland, Herston 4006, Queensland, Australia; Faculty of Medicine, University of Queensland, Herston 4006, Queensland, Australia; Infectious Diseases Unit, Royal Brisbane and Women's Hospital, Herston 4029, Queensland, Australia; Saw Swee Hock School of Public Health, National University of Singapore, Singapore 117549

**Keywords:** machine learning, nasopharyngeal swab, *Pneumocystis jirovecii* pneumonia, predictive models

## Abstract

**Background:**

The global burden of the opportunistic fungal disease *Pneumocystis jirovecii* pneumonia (PJP) remains substantial. Polymerase chain reaction (PCR) on nasopharyngeal swabs (NPS) has high specificity and may be a viable alternative to the gold standard diagnostic of PCR on invasively collected lower respiratory tract specimens, but has low sensitivity. Sensitivity may be improved by incorporating NPS PCR results into machine learning models.

**Methods:**

Three supervised multivariable diagnostic models (random forest, logistic regression and extreme gradient boosting) were constructed and validated using a 111-person Australian dataset. The predictors were age, gender, immunosuppression type and NPS PCR result. Model performance metrics such as accuracy, sensitivity, specificity and predictive values were compared to select the best-performing model.

**Results:**

The logistic regression model performed best, with 80% accuracy, improving sensitivity to 86% and maintaining acceptable specificity of 70%. Using this model, positive and negative NPS PCR results indicated post-test probabilities of 84% (likely PJP) and 26% (unlikely PJP), respectively.

**Conclusions:**

The logistic regression model should be externally validated in a wider range of settings. As the predictors are simple, routinely collected patient variables, this model may represent a diagnostic advance suitable for settings where collection of lower respiratory tract specimens is difficult but PCR is available.

## Introduction

*Pneumocystis jirovecii* is an opportunistic fungal pathogen causing pneumonia in immunocompromised hosts, such as those with human immunodeficiency virus (HIV)/acquired immunodeficiency syndrome (AIDS).^1^ The global burden of *P. jirovecii* pneumonia (PJP) remains considerable due to the increasing use of immunosuppressive agents for various indications such as transplantation, cancer and autoimmune disorders in both high- and low- and middle-income countries (LMICs), alongside suboptimal control of the HIV/AIDS epidemic in the latter.^2^

In many countries, polymerase chain reaction (PCR) on lower respiratory tract specimens such as induced sputum or broncho-alveolar lavage fluid is the gold standard diagnostic method for PJP.^3^ However, obtaining these specimens is invasive and requires costly equipment and facilities, along with skilled staff. This is particularly problematic in resource-poor settings, such as those in developing countries and rural and remote areas. Even in high-income countries, the performance of procedures to obtain lower respiratory tract specimens is associated with unintended morbidity and mortality and may not be tolerated by the elderly, the very young and the very hypoxic.^4^ We have previously reported the feasibility of nasopharyngeal swab *P. jirovecii* PCR as an alternative, less invasive diagnostic method in adults, finding it to have high specificity (1.0) but low sensitivity (0.66).^5^

The incorporation of nasopharyngeal swab *P. jirovecii* PCR in machine learning predictive models with other clinically relevant, routinely collected patient variables may be able to increase the sensitivity to levels acceptable for patient care while maintaining adequate specificity. In this study, we aimed to develop and validate such models using the same dataset as our earlier work and evaluate their performance as clinical diagnostic tools for PJP.

## Methods

### Data source

The data for this study were obtained from a state-wide computerized database (AUSLAB; Citadel Health, Canberra, Australia) containing results of all laboratory investigations requested in Queensland public sector healthcare facilities. This database also contains relevant patient demographic and clinical details.

### Setting and participants

Queensland is a large Australian state with an area of 1 730 648 km^2^ and a multi-ethnic population of 5 378 277 as of 31 December 2022.^6,7^ The estimated HIV prevalence was 0.14% as of 31 December 2018.^8^

Eligible patients during the period 1 January 2015–31 December 2016 were identified from the AUSLAB database. These patients had to meet all three eligibility criteria: clinically suspected PJP, *P. jirovecii* PCR performed on lower respiratory tract specimens (either induced sputum or broncho-alveolar lavage fluid) over 2 y from 1 January 2015 to 31 December 2016 and *P. jirovecii* PCR performed on nasopharyngeal swabs collected by healthcare staff within 7 d of lower respiratory tract specimen collection.

Demographic, clinical and laboratory data from the 111 patients who met the inclusion criteria for this study, and which constituted the dataset, are shown in Table 1. The *P. jirovecii* PCR method used is described in an earlier publication.^5^

**Table 1.** Participant characteristics.

Characteristics	Patients without PJP (n = 40)	Patients with PJP (n = 71)
Age (years), median (IQR)	62 (46–73)	66 (55–72)
Sex, n (%)		
Male	19 (17.1)	32 (28.8)
Female	21 (18.9)	39 (35.1)
Immunosuppression type, n (%)		
Haematological malignancy or transplant	14 (12.6)	13 (11.7)
Solid organ malignancy	7 (6.3)	28 (25.2)
Solid organ transplant	7 (6.3)	3 (2.7)
HIV positive	1 (0.9)	11 (9.9)
Other iatrogenic immunosuppression	4 (3.6)	14 (12.6)
Other non-iatrogenic immunosuppression	7 (6.3)	2 (1.8)
Nasopharyngeal swab *P. jirovecii* PCR result, n (%)		
Not detected	40 (36.0)	24 (21.6)
Detected	0 (0.0)	47 (42.3)

IQR: interquartile range.

Patients with PJP were those who were clinically suspected of having the disease and had positive *P. jirovecii* PCR results on lower respiratory tract specimens (induced sputum or broncho-alveolar lavage fluid).

### Outcome measure and model predictors

The outcome measure was PJP confirmed by detection of *P. jirovecii* on lower respiratory tract specimens. The predictors in the machine learning models were age (in years), gender (male vs. female), immunosuppression type (HIV, solid organ malignancy, solid organ transplant, haematological malignancy or transplant, other iatrogenic immunosuppression and other non-iatrogenic immunosuppression) and *P. jirovecii* PCR result on a nasopharyngeal swab (detected vs. not detected). These predictors were chosen to enable the models to be as simple and generalizable as possible, given the size of the dataset.

### Statistical analysis

Patients for whom nasopharyngeal swab *P. jirovecii* PCR results were unavailable were excluded from the analysis.

The baseline upon which to compare the performance of the machine learning models was the raw diagnostic sensitivity (0.66) and specificity (1.0) as well as the positive and negative predictive values (PPV 1.0 and NPV 0.63) of nasopharyngeal swabs for *P. jirovecii* PCR, which we have previously reported.^5^

Three types of supervised machine learning predictive models, namely random forest, logistic regression and extreme gradient boosting, were constructed and their performances compared. These techniques were selected as they are the most widely used for classification problems.^9^ The maximum tree depth and maximum number of features per tree in the random forest model were tuned to 4 and 9, respectively, and the maximum number of trees at 252. The learning rate (shrinkage) in the extreme gradient boosting model was tuned to 0.1, the maximum tree depth to 7 and the maximum number of trees to 256. The logistic regression model used a newton-cg solver and was regularized using Ridge (L2) regression with a C-value of 0.3.

Repeated stratified 10-fold cross-validation was used to minimize overfitting and evaluate the models. The diagnostic sensitivity, specificity, PPV and NPV of each model were calculated. The other measures used in evaluating the performance of each model on test data were its accuracy, area under the receiver operating characteristic curve (AUROC), F1 score (a summary metric combining PPV and sensitivity with equal weighting on both), F2 score (a summary metric that weights sensitivity higher than PPV) and Brier score (a summary metric of the error in the model predictions).^10,11^

Finally, positive and negative likelihood ratios and the effect in this patient cohort on post-test probability of positive and negative results from the models, as well as from using nasopharyngeal swabs for *P. jirovecii* PCR singly, were calculated from the sensitivity and specificity values to demonstrate the applicability of these four modalities as clinical diagnostic tools.

Analyses were performed using Python version 3.10 (https://www.python.org/). This report was prepared in accordance with the Transparent Reporting of a multivariable prediction model for Individual Prognosis Or Diagnosis (TRIPOD) guideline.^12^ The completed TRIPOD checklist can be found in Supporting Information S1.

## Results

### Machine learning model performance

Model performance measures are shown in Table 2.

**Table 2.** Machine learning performance measures.

Variable	Random forest model (mean±SD)	Logistic regression model (mean±SD)	Extreme gradient boosting model (mean±SD)
Accuracy	0.73 ± 0.14	0.80 ± 0.12	0.73 ± 0.13
Sensitivity	0.76 ± 0.18	0.86 ± 0.14	0.80 ± 0.16
Specificity	0.68 ± 0.26	0.70 ± 0.22	0.60 ± 0.26
PPV	0.82 ± 0.13	0.84 ± 0.10	0.79 ± 0.12
NPV	0.65 ± 0.23	0.77 ± 0.21	0.65 ± 0.25
AUROC	0.84 ± 0.13	0.89 ± 0.10	0.85 ± 0.13
F1 score	0.77 ± 0.12	0.84 ± 0.10	0.78 ± 0.11
F2 score	0.76 ± 0.15	0.85 ± 0.12	0.79 ± 0.13
Brier score	0.15 ± 0.06	0.14 ± 0.03	0.18 ± 0.08

SD: standard deviation.

Ranges for all measures are between 0 and 1. Better model performance is indicated by higher values for accuracy, AUROC, F1 score and F2 score and lower values for Brier score. The AUROC indicates the performance of the model in discriminating between outcomes. The F1 score is a summary metric combining PPV and sensitivity with equal weighting on both, while the F2 score weights sensitivity higher than PPV. The Brier score is a summary metric of the error in the model predictions.

### Clinical application

The pre-test probability in the study patient cohort of having PJP was 0.64 (number of patients with PJP/total number of patients). The positive and negative likelihood ratios of using nasopharyngeal swab *P. jirovecii* PCR as a sole predictor, the random forest model, the logistic regression model and the extreme gradient boosting model were ∞ and 0.34, 2.38 and 0.35, 2.87 and 0.20, and 2.0 and 0.33, respectively. The post-test probabilities calculated by applying these likelihood ratios to the pre-test probability are shown in Table 3.

**Table 3.** Post-test probabilities of having PJP based on the results of the diagnostic modalities in this study.

Result	Post-test probability			
	Nasopharyngeal swab *P. jirovecii* PCR only	Random forest model	Logistic regression model	Extreme gradient boosting model
Positive	1.0	0.81	0.84	0.78
Negative	0.38	0.39	0.26	0.37

The pre-test probability of having PJP was 0.64 based on the study patient cohort. Post-test probability ranges and their corresponding likelihoods of disease are >0.9, very likely; 0.67–0.90, likely; 0.34–0.66, uncertain; 0.10–0.33, unlikely; and <0.10, very unlikely.^13^

All three machine learning models increased the sensitivity for PJP from 0.66 using nasopharyngeal swab *P. jirovecii* PCR as a sole predictor to >0.75 (Tables 2 and 3). However, only the logistic regression model resulted in an acceptable loss of specificity, thus performing best out of the three machine learning models and should be preferred for external validation. Importantly, the post-test probability of a positive nasopharyngeal swab *P. jirovecii* PCR result (0.84) can be interpreted as ‘likely PJP’ and a negative result (0.26) as ‘unlikely PJP’ as per Power et al.^13^ In the other modalities, while a positive result still indicated a patient was likely to have PJP, the meaning of a negative result was uncertain.

## Discussion

In this study, we showed that incorporating a nasopharyngeal swab *P. jirovecii* PCR result as a predictor in a supervised logistic regression machine learning algorithm using only three other readily available variables was able to diagnose PJP with good accuracy among immunocompromised patients in a low HIV prevalence setting. This model also performed well when metrics such as the AUROC, F1 and F2 scores and Brier score were considered, highlighting its suitability for further evaluation as an alternative to the invasive gold standard of collecting lower respiratory tract specimens.

From a clinical perspective, the model improved diagnostic performance compared with using nasopharyngeal swab *P. jirovecii* PCR alone, as it substantially increased sensitivity without excessively compromising specificity. This is important because the pre-test probability of PJP of 0.64 indicates a considerable degree of uncertainty, thus for a diagnostic tool to be widely useful in this context it should change the post-test probability sufficiently to make the disease likely or very likely when a positive prediction is returned, and vice versa. While using nasopharyngeal swab *P. jirovecii* PCR alone may be adequate to confirm the diagnosis if *P. jirovecii* is detected, a negative test yields a post-test probability of 0.38, which is insufficient to rule out a false negative result. In contrast, while the logistic regression model has a lower specificity of 0.70, it has a much higher sensitivity (0.86) than nasopharyngeal swab *P. jirovecii* PCR alone. Therefore, a positive prediction using this model gives a post-test probability of 0.84, indicating that PJP is likely (post-test probability 0.67–0.90) while a negative prediction gives a post-test probability of 0.26, indicating that PJP is unlikely (post-test probability 0.10–0.33).^13^

To our knowledge, this is the first study supporting the use of *P. jirovecii* PCR on nasopharyngeal swabs combined with patient demographic and clinical predictors in a supervised machine learning model as a diagnostic tool for PJP in immunosuppressed adult patients living in a low HIV prevalence setting. The algorithm is simple and can be easily deployed on mobile devices and computer workstations as an app, facilitating its use in healthcare facilities. Since access to mobile devices is widespread even in LMICs, such an app would represent a valuable addition to the relatively underdeveloped digital health technology space in low-resource settings. It would also be an improvement over less sophisticated simple scoring tools, thus making good use of the computing power of these mobile devices.

Our findings are timely, given that non-HIV-infected patients have a relatively higher mortality rate from PJP compared with HIV-infected patients, underscoring the need for timely and accurate diagnosis that may be hindered by delays collecting lower respiratory tract specimens.^14^ Our results also represent an advance over the one other published machine learning diagnostic model for PJP, which is more complex and requires more sophisticated laboratory and radiological investigations not always readily available, including some not routinely used in clinical practice, when diagnosis is most urgent.^9^ In contrast, apart from *P. jirovecii* PCR on nasopharyngeal swabs, no other laboratory test result is required for our model, which has broadly similar performance metrics.

Our study has several limitations. First, the results are not generalizable to children. Second, the number of patients was small and the data were observational in nature, carrying a risk of bias. Third, the dataset size limited our ability to hold out a proportion of patients as an unseen test set without compromising model development. Nevertheless, we have appropriately minimized model overfitting and performed repeated cross-validation, thus we expect the algorithm to replicate its performance with unseen adult patients from Queensland and in similar low HIV prevalence settings.

Future work should focus on externally validating and improving the model prospectively on a larger scale in a wider range of settings, including those with high HIV prevalence, and with specimens collected on the same day under optimal conditions. The latter is especially important for nasopharyngeal swabs, as poor-quality specimens carry the risk of false negative results, as we have previously shown.^5^ Additional predictors such as physiological measurements, *P. jirovecii* prophylaxis use, physical signs and symptoms or blood test results could also be considered to improve model accuracy and other performance metrics, although only variables that are easily objectively collected as part of routine care should be used as predictors to ensure optimal generalizability of the model. Similar work should also be considered in paediatric patients as well as younger adult patients, given that the median age of our study cohort was >60 y.

## Supplementary Material

ihae052_Supplemental_File

## Data Availability

The data that support the findings of this study are available upon request to the corresponding author. The data are not publicly available due to privacy or ethical restrictions.
